# A comparative study of the efficacy and functionality of 10 commercially available wrist-hand orthoses in healthy females during activities of daily living

**DOI:** 10.3389/fresc.2022.1017354

**Published:** 2022-11-01

**Authors:** Alejandra Aranceta-Garza, Karyn Ross

**Affiliations:** ^1^ Department of Biomedical Engineering, University of Strathclyde, Glasgow, United Kingdom; ^2^ Centre for Medical Engineering Technology, University of Dundee, Dundee, United Kingdom

**Keywords:** wrist-hand orthosis, activities of daily living (ADLs), functionality, motion control, impact on time, wrist splint

## Abstract

**Objective:**

Optimal wrist/hand function facilitates the performance of activities of daily living (ADL), which are associated with independent living and increased quality of life. Rheumatological, musculoskeletal, and neurological conditions or injuries can negatively impact hand/wrist function, with wrist-hand orthoses (WHOs) being prescribed to control motion and improve wrist alignment whilst enhancing hand/wrist functionality. The objective of this follow-up study was to quantify and assess the efficacy and functionality of 10 commercially available WHOs during five ADLs.

**Design:**

Randomised comparative functional study of the wrist/hand with and without WHOs.

**Participants:**

Ten right-handed healthy female participants with no underlying condition or pain affecting the wrist/hand that could influence their ability to undertake ADLs.

**Main outcome measures:**

The primary outcome was ascertaining the impact of each WHO during five ADLs. Movement was quantified in sagittal, coronal, and transverse planes with and without WHO use. The resting position, maximum mean flexion, extension, pronation, supination, and radial and ulnar deviation attained were quantified, with the time spent in wrist flexion, wrist flexion and ulnar deviation, wrist extension >15°, and radial deviation recorded. Finally, the time to complete each task was compared between conditions.

**Results:**

At rest, four WHOs maintained the desired sagittal plane wrist position, with only one preventing radial deviation with variation observed in the transverse plane. All WHOs reduced mean maximum flexion, with only 10 out of 50 tests (20%) showing a successful restriction of flexion (*p* < 0.05) and 14 out of 50 (28%) showing a reduction of the time spent in flexion (*p* < 0.05). In 42 out of 50 tests (84%), the wrist was extended >15° for a significant amount of time (*p* < 0.05), with the wrist in radial deviation in 98% for a significant amount of time (*p* < 0.001). The wrist was flexed and in ulnar deviation for a significant time for 6 out of 50 tests (12%, *p* < 0.05), whilst all WHOs impacted transverse movement, with 27% reducing it significantly, and all tasks took a longer time to complete, with 46% taking a significantly longer time (*p* < 0.05).

**Conclusion:**

The WHOs did not control movement sufficiently to successfully manage any condition requiring motion restriction associated with pain relief and were found to increase the time to complete the ADLs. Multifactorial design aspects influenced functionality, and there is a clear need for WHO redesign.

## Introduction

Optimal hand and wrist function may be affected by rheumatological, musculoskeletal, and neurological conditions or injuries, which consequently can negatively impact independent living and restrict carrying out activities of daily living (ADLs) due to painful inflammation of the joints, muscle weakness, joint stiffness, fatigue, and reduced grip strength (1).

Importantly, the prevalence of work-related musculoskeletal disorders of the hand and wrist constitutes a significant burden not only to the individual but also to society and healthcare systems globally (2). For example, in 2001 in North America, as reported to the US Bureau of Labor Statistics, of the 355,344 cases of injury or illness of the upper limb, 33,431 were sprains or strains of the hand, wrist, or fingers, with 26,794 cases of carpal tunnel syndrome (CTS) and 4,896 cases of tendinitis of the wrist/hand (3). Importantly, CTS was associated with the highest median days away from work (25 days) (3). In addition, there is an important global rheumatoid arthritis (RA) prevalence, with an incidence of 1.46% in North America, 0.80% in Africa, and 0.53% in Europe, with some country-specific data showing an incidence as high as 2.7% in Cuba, 1.8% in Lesotho, and 0.92% in Lithuania (4). Whilst the wrist and hand are commonly affected in the early stages of RA, 85% of people report hand involvement in the later stages (5, 6).

A wrist-hand orthosis (WHO) is an externally applied device that is used to modify the structural and functional characteristics of the neuromuscular and skeletal systems (7). Functional WHOs, which hold the wrist in an improved position, are commonly prescribed for people presenting with wrist and hand dysfunction and are further prescribed for prophylactic use in the workplace and during sporting activities. Typically, a WHO should only provide control of the wrist by positioning the wrist in a functional position to increase the mechanical advantage of the finger flexors, whilst improving grip strength and reducing pain associated with synovitis, if present. Although not conclusive, there is strong evidence in the literature that maximum power grip strength is achieved with the wrist in a position of extension between 15° and 30° (8, 9), with a position of up to 15° of ulnar deviation in the coronal plane (8, 10). However, if the WHO positions the wrist at a greater degree of extension (>15°), it may have a negative impact on carrying out many ADLs. It has also been demonstrated that a combination of flexion and ulnar deviation, in addition to being detrimental to power grip, is also associated with increasing levels of pain (6). Hence, in the sagittal plane, a WHO should position the wrist at 10°–15° of extension, preventing any movement towards flexion and enabling free finger movement to facilitate hand function during ADLs (11, 12). Specifically with RA, the wrist may assume a position of radial deviation with an associated ulnar drift of the fingers, which further negatively impacts hand function and grip strength, so the WHO should also aim to control the wrist radial deviation, a design feature that is rarely integrated into the design of prefabricated WHOs. There is limited evidence in the literature regarding the ideal resting position of the wrist in the transverse plane, with clinicians often aiming for a neutral position. It has been shown that in the presence of acute injuries of the distal radius and ulna, supination and pronation of the forearm should be limited (11).

Previous qualitative research explored the role of WHOs during work and recreational activities (12), with participants reporting that the WHOs helped to increase function, although there was no quantitative assessment undertaken to support this. Previous studies have investigated the impact on performance during everyday functional tasks in the RA population (13–18). However, there is a variability seen across the results, which may be attributable to several factors such as the following: inconsistencies and/or incomplete reporting of the methodology (19), participant characteristics, WHO/s tested, the contour and fit of the WHO in the palmar region impeding grip patterns and the ability to perform ADLs, and the reporting of wrist motion control, which is often suboptimal and could be related to the design of WHOs (20).

ADLs rarely use the full wrist range of motion or require maximum grip strength to be achieved. Typically, performing an ADL requires a unique and distinct combination of motion, grip pattern/s, and grip strength. This work is a follow-up study, with previous work investigating the efficacy and functionality of 10 commercially available prefabricated WHOs with a specific emphasis on the range of motion of the wrist and grip strength (20). In this research, the impact of the same 10 WHOs on the identical population whilst performing a set of five ADLs was considered to provide a better understanding of the efficacy and functionality of prefabricated WHOs. As detailed, WHOs should maintain the wrist in a functional position of 10°–15° extension at rest and during ADLs, and crucially, it should further prevent the combination of flexion and ulnar deviation, whilst still allowing the ADLs to be completed.

## Materials and methods

Similar to previous work exploring the impact of 10 commercially available WHOs on wrist range of motion and grip strength (20), a repeatable and comprehensive testing protocol was developed to investigate the impact of 10 WHOs during a set of five ADLs: ADL1, pouring water from a jug into a cup; ADL2, turning a key in a lock; ADL3, cutting a putty block with a knife; ADL4, zipping up and down a jacket; and ADL5, moving a laden plate. The order of the WHOs to be tested, order of the condition (with/without the WHOs), and sequence of the ADLs were randomised for each individual and followed the Standards for Quality Improvement Reporting Excellence 2.0 (21).

The impact that each WHO had during the performance of the tasks was assessed by comparing, with and without the WHOs, the starting resting position, maximum mean flexion, extension, supination, pronation, radial, and ulnar deviation attained, and time to complete each task. There was a specific interest in the ability of a WHO to prevent movement into flexion or radial deviation, and if these movements occurred, the length of time when these positions were undertaken was quantified to assess the overall impact of these. Further, there was a specific interest in assessing and quantifying the amount of wrist extension sustained over 15°, the amount of wrist flexion and ulnar deviation, and the time each participant spent in wrist flexion, wrist radial deviation, wrist >15° of extension, and a combination of wrist flexion with ulnar deviation with each WHO during each ADL.

### Subjects

It was important to test each functionality of the WHOs in the absence of pain that could limit the assessment of their functionality and efficacy, and as such, 10 healthy right-handed (as confirmed by the Edinburgh handedness test) female participants (aged 36 ± 10.8) were recruited. The upper limb strength of female participants has been demonstrated to be 40%–70% less compared with their male equivalents (22). For this reason, it was deemed that healthy female participants would apply a deforming force to the WHOs that would be representative of the upper limit of the deforming forces that could be applied to the WHO by either gender presenting with wrist and hand dysfunction (20).

Exclusion criteria included subjects undertaking upper body/limb training during the test period; any musculoskeletal or neurological disorder affecting the upper limb; any injury affecting the hand, wrist, and/or arm; and any previous upper limb surgery.

### Design

A controlled, systematic, and repeatable (with all inter-WHO correlations >0.703) study was developed to evaluate the efficacy and functionality of 10 commercially available prefabricated WHOs during five ADLs. This study ran over a period of 10 weeks, where each participant randomly (computer-randomisation) tested a different WHO each week. The order of with/without a WHO condition and ADL was also computer-randomised. Participants’ height and weight were recorded every week throughout the test period, as there is a correlation between weight and grip that could account for any effects due to confounding factors that may impact the results.

### Hardware and configuration

Each participant was instrumented with a two-axis electro-goniometer (SG65, Biometrics Ltd., UK) that was used to measure wrist flexion/extension and radial/ulnar deviation in the sagittal and coronal planes, respectively, with a torsiometer positioned to measure wrist/forearm pronation/supination. A second electro-goniometer was used to measure elbow flexion/extension, which was used to identify the start and end of each ADL. All sensors were securely attached using a double-sided medical grade tape and an elasticated stockinet and were positioned by the same researcher across all participants and testing sessions. In addition, to minimise positional uncertainties, each unit was manually checked using a traditional mechanical goniometer and zeroed accordingly before the start of every testing session.

### Weekly testing protocol

The 10 prefabricated WHOs that were selected for testing (Table 1) reflected variations in the commercially available designs with regard to geometry, materials of construction, and fastenings.

**Table 1:** Range of commercially available wrist-hand orthoses used in this study showing their length (in cm), construction material, fastenings, the type of volar bar, and the presence of an additional wrap around the wrist strap.^a^

ID	Length (cm)	Construction material and fastenings	Volar bar material	Wrist strap	Image
1	23	Two-way stretch fabric and Velcro® fastenings	Aluminium	N	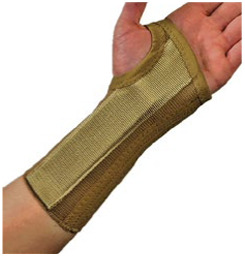
2	23	Neoprene with Velcro® fastenings	Aluminium	N	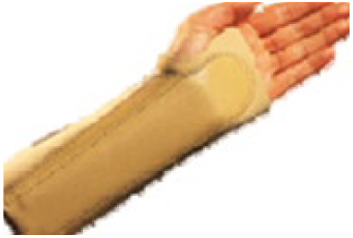
3	23	Two-way stretch fabric and Velcro® fastenings	Aluminium	Y	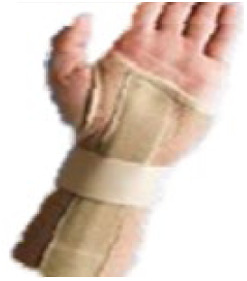
4	25	Silicone and Velcro® fastenings	Plastic	N	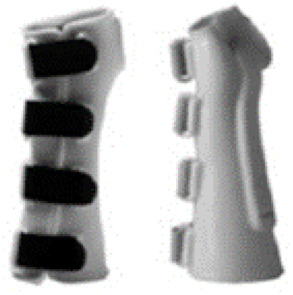
5	20	Neoprene with dorsal plastic stays and Velcro® fastenings	Aluminium	N	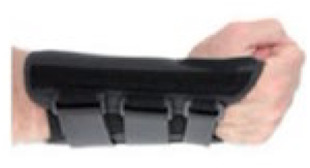
6	18	Two-way stretch fabric and Velcro® fastenings	Aluminium	N	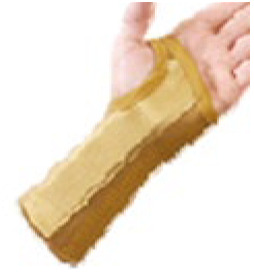
7	25	Neoprene with dorsal plastic stays and Velcro® fastenings	Aluminium	N	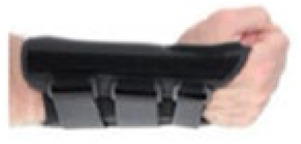
8	20	Fabric type, single lace, and/or Velcro® fastenings	Aluminium	N	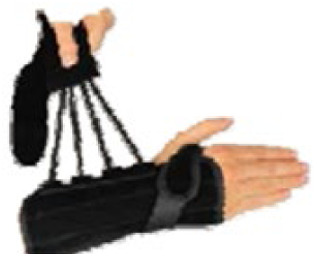
9	20	Neoprene with a plastic pocket and Velcro® fastenings	Aluminium	N	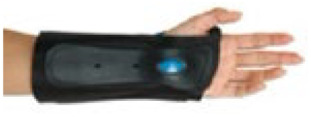
10	18	Neoprene and Velcro® fastenings	Aluminium	N	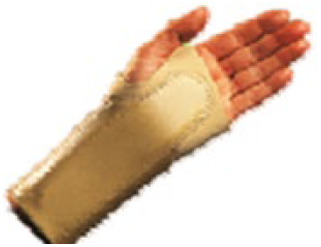

^a^ From (20).

Each WHO was fitted to each participant according to the manufacturer's guidelines by the same expert team, comprising an orthotist and sports engineer throughout the duration of the study, to ensure consistency and also that the optimal fit of each WHO was achieved. Although some of the WHOs tested had a removable aluminium volar bar, this contour was not altered to change the alignment of the wrist section of the device in the sagittal plane prior to fitting, as a commissioned qualitative study undertaken by the researchers for Versus Arthritis UK has indicated that the volar bar is infrequently adjusted. The angle of the volar bar was measured before and after testing to check for any deformation or changes in its sagittal plane alignment as a result of the activity.

Five ADLs were chosen to reflect differing grip patterns and activities likely to be performed every day. All participants had the same defined starting and finishing positions; outside of this, the participants were free to move as necessary to complete the activity. Each ADL was demonstrated to the participant with a single practice session undertaken before recording their movement during each ADL. The participants had a 2-minute resting period between each ADL, with each one being carried out only once to prevent any learning effect.

For the starting/finishing position:

• Participant standing in front of the test table;
• Shoulders adducted at 90° and neutrally rotated (confirmed by the manual goniometer at the start of each test);
• Elbow fully extended;
• Forearm in neutral pronation/supination;
• Wrist in neutral flexion/extension; and
• Wrist in neutral radio/ulnar deviation.

**Figure 1:**
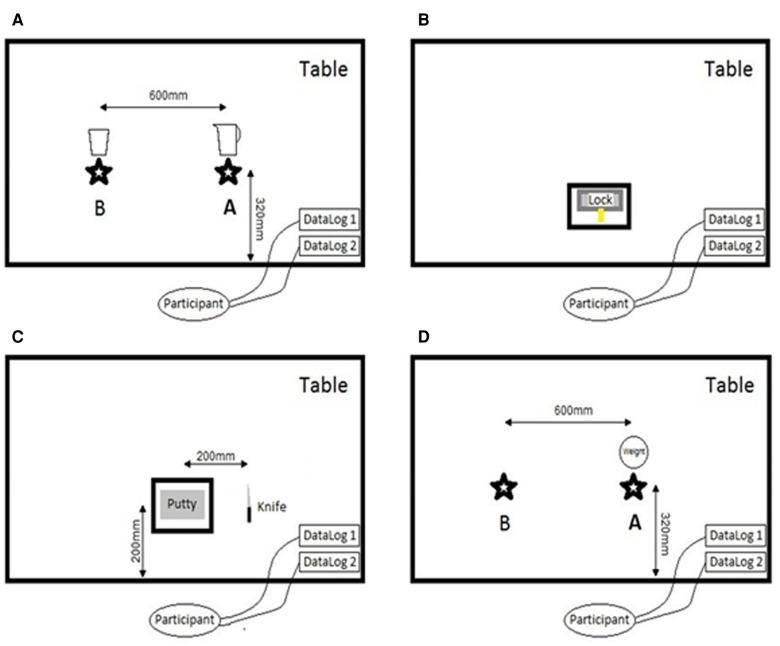
Example of a setup for ADL1 (**A**), ADL2 (**B**), ADL3 (**C**), and ADL4 (**D**).

Activities of daily living investigated:

*ADL1: Pouring water from a jug into a cup*—This ADL requires a stable wrist in the sagittal and coronal planes, pronation/supination motion, and the ability to maintain a stable transverse volar power grip under the given load of the jug and water (which were kept consistent for all participants). The participants were instructed to move from the starting position, pick up the jug positioned at point A, and pour the water into the cup at point B (Figure 1A). The participants were asked not to lift the cup at point B. After filling up the cup, the participants returned the jug to point A and also returned to the starting position, indicating that the task was finished.
*ADL2: Turning a key in a mortice lock—*This ADL uses a lateral pinch and was used to measure the effect that each WHO had on tasks specifically requiring pronation/supination. From the starting position, the participants took hold of the key that was already positioned in the lock. They were instructed to turn the key fully anticlockwise, stopping once the lock was fully rotated. After a full rotation, the participants turned the key clockwise until once more the lock was fully rotated. After this, the participants returned to the starting position to indicate that the task was finished (Figure 1B).
*ADL3: Cutting a putty block with a knife*—This common kitchen-based ADL uses a diagonal volar grip and was selected to highlight a complex controlled activity requiring a stable wrist with the need for a challenging and stable grip pattern when applying a downward cutting motion/load. From the starting position, the participants were asked to pick the knife up, which was always positioned at the same distance from the putty, with the blade facing right when looking at it. After picking it up, the participants were instructed to only use their right hand for cutting, although the putty block could be supported with the left hand. They were instructed to cut the putty twice, cutting the putty into three approximately equal portions. Once completed, the participants returned the knife to the original position and then to the starting position to indicate that the task was finished (Figure 1C).
*ADL4: Zipping up and down a jacket*—This activity involves a pulp pinch and relies mainly on the wrist adopting a position of flexion, with the rest of the motion coming from the elbow and shoulder. Dressing can be an arduous task with wrist/hand dysfunction and/or pain. This activity simulates a part of the dressing process and the effect that each WHO has on the motion used and the time taken to get dressed. The participants were provided with a gilet/vest to wear during this activity. From the starting position, the participants were instructed to gather the zipper at the base and pull it up to reach the top, and then undo the zip fully before returning to the starting position to indicate that the task was finished.
*ADL5: Moving a laden plate*—Lifting a laden plate is representative of many tasks undertaken as part of an individual's activities in the workplace, at home, or during recreation. Typically, pronation/supination is required until contact is made with an extension grip on the plate, with the maintenance of a strong grip and stable wrist position in all three planes until the laden plate is released. Maintaining a strong lateral grip on a laden plate places significant stress on the wrist. From the starting position, the participants were instructed to pick up a plate with their right hand, move it from point A to B, return it to point A, and then return to the standing starting position to indicate that the task was finished (Figure 1D).

### Statistical analyses

The efficacy and functionality of each WHO during the ADLs and the impact on each ADL were measured and compared with the activity without a WHO. For each condition (with/without a WHO) and for each ADL, resting position, mean maximum flexion/extension, supination/pronation, and radial/ulnar deviation were recorded for each participant. There was a specific interest in the ability of a WHO to prevent movement into flexion, radial deviation, and a combination of ulnar deviation with wrist flexion. Further, there was a specific interest in the time each participant spent in wrist flexion, wrist radial deviation, and wrist >15° of extension with each WHO during each ADL. An additional measure of interest was the total time to complete the task with and without the WHOs. All the data were tested for normality.

#### Volar bar angle

Each WHO’s volar bar was measured before and after each session. The volar bar was not adjusted or contoured and was used as was supplied by manufacturers.

#### Resting position

A wrist extension of 10°–20° has been described as the optimal position for those users presenting with synovitis (23), as it has been shown to reduce stress on peri-articular structures, the joint capsule, and the synovial lining (24), whilst optimising the efficiency of the flexor muscles. Whilst this is important, the position should also reflect the best wrist posture for pain relief for the individual (25). There is a lack of consensus regarding the exact position of the wrist in the sagittal plane that could be associated with relieving symptoms related to carpal tunnel syndrome (often present in those with RA), which varies from slight flexion (26) to a range of 10°–15°extension (23) or neutral (27).

If the desired resting position in the sagittal plane is to be maintained during ADLs, and if any propensity to wrist radial deviation is to be addressed, then the WHO should prevent motion beyond the defined resting position, that is, the WHO should restrict any motion towards flexion and radial deviation. Therefore, and in line with the defined optimal position at rest to reduce stress on the periarticular structures (23), the resting values with the WHOs were compared with the prescribed 10°–15° of extension using a 1-sample *t*-test or one-sample Wilcoxon signed rank test (*α* = 0.05).

#### Flexion/extension, radial/ulnar deviation, and pronation/supination

As a primary objective of prescribing a WHO for RA management is to restrict wrist flexion and radial deviation, we assessed the efficacy of each WHO in restricting flexion and radial deviation and quantified it in two ways: firstly, one-sample equivalent tests (*α* = 0.05) were used to assess if the mean maximum flexion and mean maximum radial deviation were ≤0; secondly, the time spent in flexion and one-sample *t*-tests (*α* = 0.05) were used to compare the time each WHO allowed going into flexion with zero. Similarly, the time spent in radial deviation was quantified and significant differences were compared with zero (one-sample *t*-tests, *α* = 0.05). The time spent in >15° of extension was quantified and compared with zero. In the absence of a significant body of evidence that could inform the ideal restriction of pronation/supination whilst wearing a WHO, our results will describe the movement during the activities undertaken.

#### Time to complete a task

The time to complete a task was measured with and without WHOs for each participant. As the start and finish positions were the starting position, the timing started as soon as the elbow was flexed to undertake the task and stopped once the participant returned to the starting position. The times were compared with and without WHOs, and Wilcoxon matched-pairs signed-rank tests (*α* = 0.05) were used to assess if the mean difference was statistically different from zero.

## Results

There were a total of ten healthy right-handed female participants with a height of (1.63 ± 0.05) m, BMI of (25.39 ± 4.40) kg/m^2^, weight of (67.16 ± 13.84) kg, and a maximum grip strength of (26.39 ± 4.40) kg. The participants' weight did not vary more than 2 kg throughout the sessions.

### Resting position

**Figure 2:**
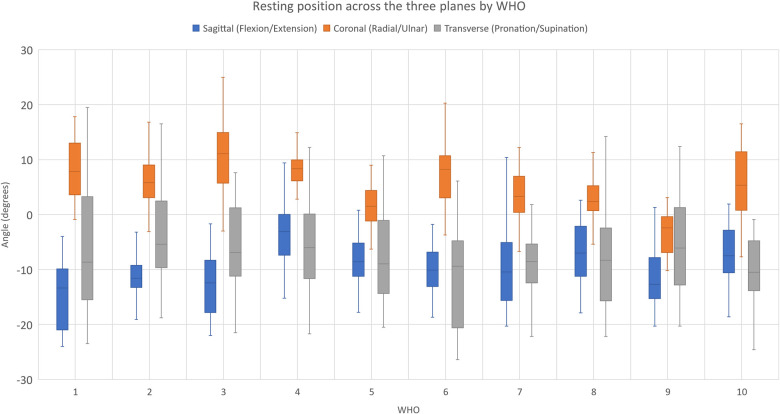
Resting position across the three planes by WHOs across ADLs. For the coronal plane, statistical significance is denoted by+and ++ when the mean value was statistically greater than 0, indicating that the wrist was at radial deviation with *p* < 0.05 and *p* < 0.001, respectively. For the sagittal plane, statistical significance is denoted by * and ** when the resting value was statistically different from the prescribed 10°–15° of extension for *p* < 0.05 and *p* < 0.001. The red dotted lines show the prescribed resting positions in the sagittal plane (10° and 15° of extension).

In Table 2 and Figure 2, the mean (SD) resting positions for the three planes (sagittal, coronal, and transverse) across all ADLs by WHOs are shown.

**Table 2:** Resulting mean (SD) resting position in the three planes (sagittal, coronal, and transverse) by WHOs across all ADLs.

WHO	Resting position with WHOs		
	Sagittal (F/E)	Coronal (R/U)	Transverse (P/S)
	Mean (SD) (°)	Mean (SD) (°)	Mean (SD) (°)
1	−7.62 (4.74)	8.27 (5.24)++	−5.77 (13.30)
2	−8.49 (5.02)	6.56 (4.46)++	−1.87 (12.02)
3	−4.79 (8.27)	10.41 (6.77)++	−5.75 (7.29)
4	−1.25 (6.05)**	7.66 (4.92)++	−10.42 (19.93)
5	−4.91 (4.34)*	1.49 (3.97)+	−7.26 (8.40)
6	−8.87 (5.13)	7.62 (5.73)++	−10.23 (10.95)
7	−2.62 (7.18)**	3.00 (5.81)+	−8.78 (6.80)
8	−6.91 (4.16)**	2.96 (4.57)++	−8.48 (8.42)
9	−3.66 (4.97)	−3.43 (3.62)	0.26 (21.59)
10	−6.82 (5.58)**	5.88 (6.00)++	−4.32 (22.24)

For the sagittal plane (flexion/extension), statistical significance when the mean was outside the optimal resting values of 10° and 15° (* and ** denote *p* < 0.05 and *p* < 0.001, respectively). Shaded in grey are those WHOs that did stay within the desired range in the sagittal and coronal planes at rest. For the coronal plane (radial/ulnar deviation), statistical significance is shown when the mean value was statistically greater than 0, allowing for radial movement.+and ++ denote *p* < 0.05 and *p* < 0.001, respectively. For the transverse plane (pronation/supination), resting positions are reported. The negative numbers imply that the wrist is in extension, ulnar deviation, or supination.

It can be seen from Table 2 and Figure 2 that at rest, WHO#1, #2, #3, #6, and #9 maintained the wrist within the desired resting position range in the sagittal plane, with only WHO#9 preventing a resting position of radial deviation and significant variation across the transverse plane.

#### Flexion/extension movement

Maximum flexion and extension movements were obtained and compared with and without a WHO for each ADL (paired sample *t*-tests or Wilcoxon signed rank tests, accordingly), and the time spent in flexion with a WHO was quantified. Each WHO’s ability to block flexion was estimated using one-sample equivalent tests (mu = 0) when the maximum mean flexion was ≤0. The statistical tests are reported in Table 3.

**Table 3:** Mean maximum flexion with and without WHOs by ADLs.

WHO	ADL1		ADL2		ADL3		ADL4		ADL5	
	Mean (SD) (°)		Mean (SD) (°)		Mean (SD) (°)		Mean (SD) (°)		Mean (SD) (°)	
	Without WHO	With WHO	Without WHO	With WHO	Without WHO	With WHO	Without WHO	With WHO	Without WHO	With WHO
1	34.49 (9.60)	−6.71 (5.37)** ++	11.96 (9.66)	−1.71 (15.55)**	13.95 (10.64)	4.43 (16.70)	31.52 (11.97)	0.73 (9.18)**	33.57 (11.69)	−10.90 (6.03)** ++
2	32.96 (20.91)	−3.05 (4.42)**	11.30 (8.84)	−4.17 (3.97)** +	26.01 (12.96)	−3.17 (4.57)**	32.22 (20.66)	6.38 (8.97)*	34.19 (11.19)	−6.97 (5.71)** +
3	19.34 (16.25)	−0.51 (17.61)*	13.07 (13.97)	−3.11 (9.72)	25.79 (18.55)	1.03 (8.72)*	44.57 (16.99)	3.52 (7.36)**	25.75 (13.70)	−8.61 (6.06)** +
4	37.37 (9.64)	−1.42 (7.38)**	15.76 (6.29)	0.26 (7.75)**	30.53 (14.69)	−0.29 (6.00)**	41.03 (19.62)	8.23 (12.59)*	32.11 (17.23)	−0.67 (5.87)**
5	32.12 (15.62)	−2.12 (3.99)**	18.90 (10.32)	−4.26 (5.48)** +	27.77 (9.25)	−0.95 (4.69)**	41.56 (16.83)	5.71 (7.00)**	35.90 (14.56)	−6.06 (5.08)** +
6	34.47 (19.73)	−0.59 (5.66)**	12.9 (10.04)	−2.27 (6.44)*	29.40 (12.18)	2.78 (7.98)**	40.48 (13.88)	10.23 (7.23)**	36.95 (17.39)	−4.29 (5.45)**
7	28.00 (13.93)	−3.88 (12.16)**	14.80 (13.10)	−1.33 (11.24)**	26.41 (15.41)	−0.65 (16.85)**	36.14 (5.48)	2.87 (7.89)**	28.89 (16.87)	−4.03 (12.23)**
8	38.65 (17.20)	−3.15 (7.60)**	15.50 (13.15)	−0.41 (6.75)*	30.31 (8.18)	−1.13 (5.83)**	45.37 (15.53)	6.28 (7.37)**	37.03 (8.10)	−4.65 (5.34)** +
9	30.67 (23.41)	−3.41 (5.47)** +	20.4 (13.36)	−3.80 (7.40)**	29.94 (17.86)	−1.76 (7.36)*	42.73 (14.16)	7.67 (5.68)**	36.73 (18.48)	−7.37 (5.71)** +
10	29.69 (17.58)	0.53 (7.41)**	17.58 (12.10)	0.69 (11.53)*	31.62 (16.23)	4.04 (10.45)**	41.81 (22.40)	10.77 (6.00)*	30.65 (14.37)	0.43 (10.77)**

Statistical significance was obtained using paired-sample *t*-tests (*α* = 0.05), with * and ** denoting *p* < 0.05 and *p* < 0.001, respectively. Statistical test with WHO ≤0 is denoted, with + and ++ denoting *p* < 0.05 and *p* < 0.001, respectively. The negative numbers imply extension. In grey are those ones that were found to be statistically significant between with and without WHOs as well as ≤0.

It can be observed from Table 3 that all WHOs reduced the mean maximum flexion throughout the ADLs when compared with without the WHOs, with only some tests (10 out of 50 tests) being significantly lower than zero, implying a successful reduction in flexion during a task.

**Figure 3:**
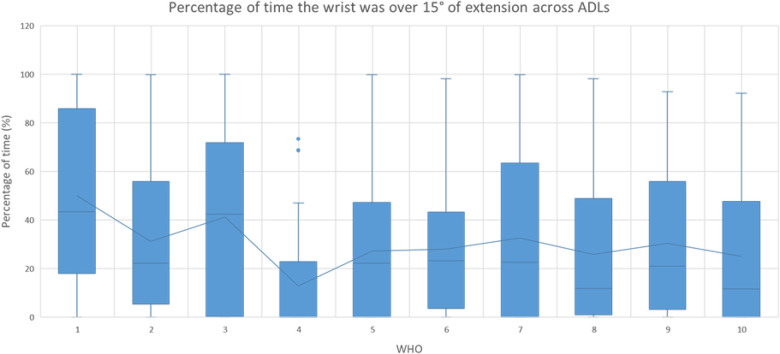
Percentage of time when the wrist adopted a flexed position throughout the activity. In the figures, the outliers are shown with the points, and each mean is shown by the lines. Statistical significance is shown by * and ** when the mean was statistically greater than 0, for *p* < 0.05 and *p* < 0.001, respectively. The mean for each group is shown by the lines, and the outliers are shown with the circles.

The percentage of time that the wrist was in a flexed position during the activities was calculated and is given in Table 4, with Figure 3 showing the percentage of time spent in wrist flexion with WHOs across ADLs (regardless of the activity). One-sample Wilcoxon signed-rank tests were used to assess if the percentage of time spent during flexion was statistically different from 0 with each WHO and by each ADL.

**Table 4:** Percentage of time that the wrist was flexed with and without WHOs during all ADLs.

WHO	ADL1		ADL2		ADL3		ADL4		ADL5	
	Mean (SD) (%)		Mean (SD) (%)		Mean (SD) (%)		Mean (SD) (%)		Mean (SD) (%)	
	Without WHO	With WHO	Without WHO	With WHO	Without WHO	With WHO	Without WHO	With WHO	Without WHO	With WHO
1	16.03 (14.56)	1.17 (3.52)+	10.16 (10.77)	4.81 (13.27)	19.66 (20.80)	3.03 (4.90)+	37.80 (20.71)	6.88 (11.98)+	16.54 (13.90)	0.00 (0.00) +
2	18.45 (22.18)	3.58 (8.58)	11.59 (16.34)	0.04 (0.14)+	19.29 (19.40)	0.86 (2.16)+	38.75 (29.45)	20.07 (19.31)*	16.04 (17.27)	0.46 (1.45)+
3	13.89 (15.64)	5.03 (8.77)	19.37 (19.18)	0.95 (1.30)+	27.30 (22.23)	6.08 (12.40)*+	48.44 (26.01)	6.92 (8.26)* +	23.18 (21.36)	0.81 (0.26)+
4	24.05 (23.53)	19.38 (30.85)*	21.06 (18.78)	9.14 (17.19)+	35.46 (19.31)	13.82 (23.73)+	52.79 (25.96)	39.00 (37.81)*	16.91 (15.40)	16.41 (29.05)
5	24.63 (24.29)	5.01 (10.27)+	22.92 (22.95)	0.40 (0.86)+	28.80 (33.21)	4.70 (14.08)+	52.10 (25.59)	14.18 (16.53)* +	28.98 (25.13)	2.00 (6.31)+
6	23.93 (20.43)	7.68 (11.59)+	24.39 (17.03)	0.34 (1.03)+	26.40 (22.01)	8.15 (13.33)+	57.09 (26.78)	25.59 (21.61)* +	25.57 (25.98)	0.92 (1.60)+
7	24.70 (30.37)	11.44 (31.29)+	27.08 (29.07)	10.53 (31.37)+	29.38 (29.22)	10.06 (31.49)+	49.95 (27.32)	26.68 (28.87)* +	22.41 (30.08)	10.12 (31.56)+
8	26.31 (20.65)	5.30 (9.54)+	22.13 (17.95)	3.47 (9.23)* +	35.28 (18.96)	3.26 (5.81)+	52.34 (31.01)	26.42 (33.98)* +	25.70 (21.85)	6.47 (13.63)+
9	24.85 (19.65)	6.28 (18.46)+	19.65 (17.48)	3.04 (8.23)+	30.68 (25.83)	3.45 (7.58)+	46.13 (19.85)	20.88 (24.09)* +	17.74 (18.84)	4.17 (12.52)+
10	24.35 (29.02)	14.97 (32.58)*	22.73 (31.37)	10.85 (31.30)+	28.61 (33.34)	12.34 (30.98)*	51.43 (25.91)	32.48 (28.27)* +	31.05 (32.56)	14.21 (32.08)+

* and ** denote *p* < 0.05 and *p* < 0.001, respectively, when the percentage of time was statistically greater than 0 with WHOs. Wilcoxon- matched rank tests were used to test if the differences between with and without WHOs were statistically different, with + and ++ denoting statistical significance for *p* < 0.05 and *p* < 0.001, respectively. Highlighted in grey are those WHOs that were statistically greater than 0.

All WHOs showed a reduction in the percentage of time that the wrist was flexed with them, when compared with that without them (Table 4), with the wrist spending a significant amount of time flexed with some WHOs. This reduction can be further observed in Figure 3, regardless of the ADL.

**Figure 4:**
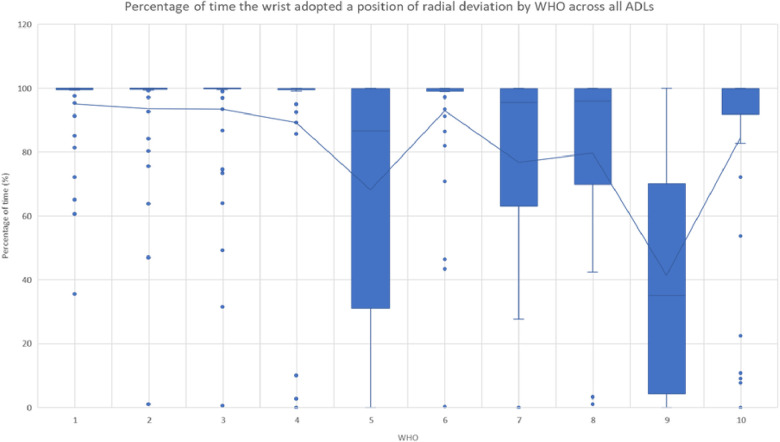
Percentage of time when the wrist was over 15° of extension regardless of the ADL by WHOs. The outliers are shown with the circles, with mean values shown by the line. Statistical significance is given in Table 5.

The time spent with the wrist over 15° of extension was quantified for each ADL by a WHO, as immobilising the wrist in a position of extension greater than this may negatively impact the ability to perform some ADLs, and movement out of this range may elicit pain. The data relating to this are presented in Table 5 for all ADLs, by WHOs, and across ADLs, with Figure 4 showing all ADLs by WHOs. One-sample Wilcoxon signed-rank tests were used to assess if the percentage of time spent over this extension range was statistically different from 0 with WHOs, by ADLs, and across ADLs.

**Table 5:** Percentage of time beyond the desired range of extension (15°) by ADLs and across all ADLs, with * and ** denoting *p* < 0.05 and *p* < 0.001, respectively, when the percentage of time was statistically greater than 0 with WHOs (one-sample Wilcoxon signed-rank tests).^a^

WHO	ADL1	ADL2	ADL3	ADL4	ADL5	ADLs
	With mean (SD) (%)	With mean (SD) (%)	With mean (SD) (%)	With mean (SD) (%)	With mean (SD) (%)	With mean (SD) (%)
1	46.29 (30.79)*	67.87 (27.22)*	45.76 (35.04)*	34.04 (38.60)*	56.06 (34.30)*	50.01 (33.86)**
2	28.33 (26.02)*	55.76 (24.18)*	19.94 (28.63)*	6.76 (6.94)	39.35 (31.34)*	31.30 (29.20)**
3	37.42 (37.51)*	55.57 (36.54)*	33.82 (37.84)*	26.54 (26.17)*	52.70 (40.74)*	41.21 (36.39)**
4	15.76 (25.02)*	32.79 (8.23)*	6.63 (3.16)	2.05 (6.16)*	7.31 (13.21)	12.91 (20.44)*
5	24.17 (25.19)*	48.08 (33.26)*	17.46 (19.35)*	10.48 (17.90)	36.66 (30.00)*	27.37 (28.25)**
6	24.84 (12.79)*	52.01 (28.00)*	15.34 (17.65)*	8.52 (13.76)	37.26 (29.27)*	28.03 (25.87)**
7	32.61 (38.63)*	49.86 (36.68)*	29.35 (35.38)*	9.61 (22.64)	42.20 (41.43)*	32.73 (36.74)**
8	27.39 (29.48)*	50.67 (32.54)**	17.95 (28.00)*	4.98 (7.02)*	27.17 (30.83)*	25.79 (30.18)**
9	32.88 (29.55)*	51.11 (23.82)*	14.73 (11.50)*	5.83 (10.09)*	49.03 (33.07)*	30.51 (28.80)**
10	26.39 (8.85)*	45.83 (9.55)*	16.01 (21.73)*	9.75 (19.96)*	27.16 (24.69)*	25.03 (27.15)**

^a^ In grey are those WHOs that are greater than 0.

In 44 out of 50 tests, the wrist was in a position of wrist extension beyond the desired range (>15°), which was statistically significant (*p* < 0.05). When all ADLs were considered together by WHOs, all tests were found to allow the wrist to go beyond this range (Table 5 and Figure 4).

#### Radial/ulnar deviation

Similar to the percentage of time spent in flexion movement, the efficacy and functionality of each WHO in restricting radial deviation was quantified by calculating the amount of time that the wrist spent in a position of radial deviation with each WHO during each ADL. One-sample Wilcoxon signed-rank tests were used to assess if the percentage of time spent with the wrist adopting a radial deviation position was statistically different from 0 for each WHO and by each ADL.

**Figure 5:**
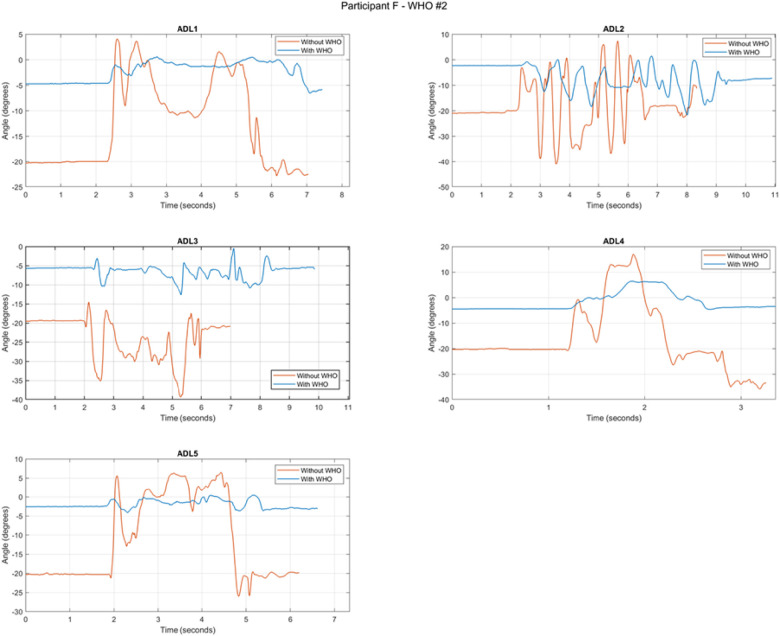
Percentage of time when the wrist adopted a position of radial deviation by WHOs across all ADLs. The outliers are shown with a circle, with the mean of each group shown by the line. Statistical significance is given in Table 6.

As seen in Table 6 and Figure 5, only WHO#9 during ADL4 was in any way effective in blocking radial deviation. However, this was not consistent across all participants.

**Table 6:** Percentage of time that the wrist was in radial deviation throughout each ADL by each WHO.

WHO	ADL1	ADL2	ADL3	ADL4	ADL5	All ADLs
	With mean (SD) (%)	With (SD) (%)	With mean (SD) (%)	With mean (SD) (%)	With mean (SD) (%)	With mean (SD) (%)
1	91.71 (14.73)**	96.22 (7.42)**	99.89 (0.10)**	91.26 (21.12)**	96.02 (11.63)**	95.02 (12.87)**
2	97.27 (6.38)**	97.44 (7.70)**	94.59 (16.69)**	78.86 (32.90)**	99.66 (0.91)**	93.34 (18.06)**
3	90.27 (22.13)**	95.30 (8.87)**	99.86 (0.30)**	84.91 (33.65)**	96.37 (11.36)**	93.34 (19.08)**
4	87.93 (31.22)**	89.66 (30.56)**	90.91 (28.42)**	87.62 (31.01)**	90.17 (30.62)**	89.26 (29.15)**
5	52.86 (38.13)*	68.17 (34.78)**	77.28 (35.41)**	68.12 (38.52)**	73.99 (40.00)**	68.08 (36.84)**
6	84.41 (32.27)**	96.57 (9.67)**	97.90 (5.80)**	84.96 (24.83)*	99.87 (0.11)**	92.92 (19.05)**
7	66.77 (36.29)**	75.80 (33.38)**	85.58 (31.95)**	70.72 (33.89)**	84.70 (33.69)**	76.72 (33.32)**
8	66.67 (38.31)**	86.74 (21.15)**	82.04 (33.05)*	79.74 (33.33)**	83.70 (31.19)**	79.73 (31.33)**
9	20.75 (16.47)*	47.96 (33.47)*	63.59 (31.55)*	21.67 (29.16)	52.74 (42.74)*	41.34 (34.97)**
10	81.04 (34.44)**	85.02 (33.17)**	87.88 (29.52)**	78.68 (36.72)**	89.77 (31.54)**	84.48 (32.06)**

* and ** denote *p* < 0.05 and *p* < 0.001, respectively, when the percentage of time was statistically greater than 0.

As it has been demonstrated previously that a combination of flexion and ulnar deviation, in addition to being detrimental to power grip, is also associated with increasing levels of pain (6), it was important to quantify the percentage of time that each WHO allowed flexion (AND) ulnar deviation for each ADL. Wilcoxon signed-rank tests were used to compare if the percentage of time spent in these positions was greater than 0 with WHOs.

As can be seen in Table 7, the combination of flexion (AND) ulnar deviation was highly task-dependent, with it occurring most during ADL4 and to a lesser degree (and depending on the WHO) during ADL1, 2, and 3. When all the ADLs were considered together, WHO#5, 7, 9, and 10 were found to allow this posture significantly (*p* < 0.05), indicating poor control of the combined motions.

**Table 7:** Percentage of time that the wrist was in flexion and ulnar deviation during each ADL and by each WHO.

WHO	ADL1	ADL2	ADL3	ADL4	ADL5	All ADLs
	With mean (SD) (%)	With mean (SD) (%)	With mean (SD) (%)	With mean (SD) (%)	With mean (SD) (%)	With mean (SD) (%)
1	0.00 (0.00)	0.37 (1.10)	0.00 (0.00)	0.17 (0.50)	0.00 (0.00)	0.11 (0.53)
2	0.00 (0.00)	0.00 (0.00)	0.65 (2.04)	4.96 (9.70)	0.00 (0.00)	1.12 (4.68)
3	0.02 (0.08)	0.09 (0.28)	0.00 (0.00)	0.85 (2.69)	0.00 (0.00)	0.19 (1.21)
4	0.00 (0.00)	0.00 (0.00)	0.00 (0.00)	2.17 (4.76)	0.00 (0.00)	0.43 (2.22)
5	3.03 (9.59)	0.22 (0.55)	4.22 (13.33)	2.87 (6.18)	2.00 (6.18)	2.47 (8.22)*
6	0.00 (0.00)	0.00 (0.00)	0.00 (0.00)	2.19 (5.68)	0.00 (0.00)	0.39 (2.35)
7	10.36 (31.51)	10.00 (31.53)	9.97 (31.51)	12.69 (30.42)*	9.99 (31.60)	10.60 (30.03)*
8	0.29 (0.09)	0.00 (0.00)	0.41 (1.29)	0.80 (1.63)	0.00 (0.00)	0.25 (0.95)
9	6.28 (18.46)	2.87 (8.29)	0.95 (2.85)	17.80 (25.34)*	4.17 (12.52)	6.42 (16.04)**
10	7.84 (24.44)	10.01 (31.53)	9.19 (29.06)	11.57 (28.33)	9.99 (31.61)	9.72 (27.92)*

* and ** denote *p* < 0.05 and *p* < 0.001, respectively, when the percentage of time was statistically greater than 0 with WHOs. In grey are those WHOs that had a time that was statistically greater than 0 for each ADL and across all of them.

#### Supination/pronation

This movement is essential when performing certain ADLs such as ADL2 (turning a key in a lock) or ADL1 (pouring water from a jug into a cup). If these movements are restricted by WHOs, as is required to reduce pain, other compensatory motions such as increased shoulder motion may be required during these types of tasks.

**Figure 6:**
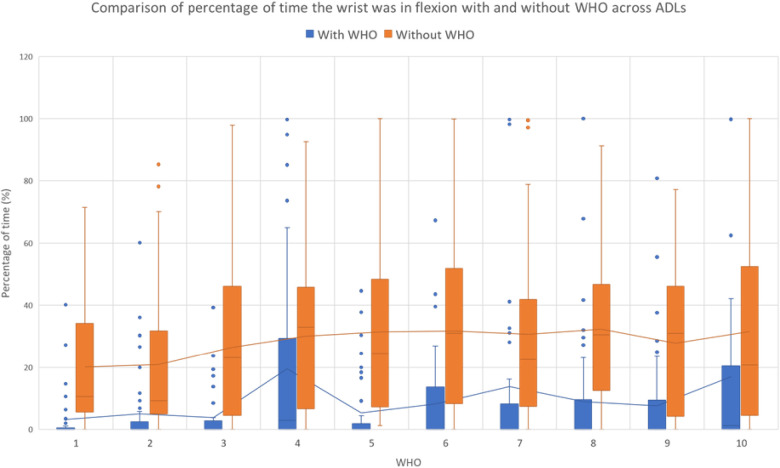
Example of a random participant's pronation and supination during each ADL with and without WHO#2. In each graph, the difference in amplitude, range of motion, and time taken to complete tasks are shown. The positive angles imply pronation, and the negative ones imply supination.

There was a reduction in pronation and supination associated with all WHOs across all ADLs, with all WHOs having some significant reduction for different ADLs (Table 8). A typical example from a random participant is shown in Figure 6, where the five ADLs are shown in the transverse plane with and without WHO. Even though there is no clinically defined prescriptive position in the transverse plane, distinctive differences can be observed in the starting position before the commencement of the task, amplitude of the range of motion, and time to complete the task.

**Table 8:** Mean maximum pronation and supination with and without WHOs by ADLs.

WHO	Motion	ADL1		ADL2		ADL3		ADL4		ADL5	
		Mean (SD) (°)		Mean (SD) (°)		Mean (SD) (°)		Mean (SD) (°)		Mean (SD) (°)	
		Without WHO	With WHO	Without WHO	With WHO	Without WHO	With WHO	Without WHO	With WHO	Without WHO	With WHO
1	P	13.60 (10.77)	4.44 (11.43)*	17.90 (10.47)	10.73 (10.59)*	−0.87 (8.82)	−0.74 (12.15)	22.46 (13.12)	7.97 (10.48)*	14.34 (9.72)	5.10 (10.20)*
	S	−19.83 (9.98)	−12.82 (16.01)	−34.10 (13.24)	−34.77 (16.22)	−29.61 (17.72)	−23.00 (13.11)	−24.66 (10.25)	−16.14 (13.03)*	−20.43 (10.77)	−13.30 (12.90)
2	P	19.29 (16.32)	12.38 (15.35)*	23.61 (18.51)	14.95 (16.24)*	5.43 (13.76)	5.67 (11.91)	26.53 (15.46)	16.65 (15.10)*	21.66 (16.80)	12.88 (17.10)*
	S	−15.93 (12.60)	−2.65 (10.60)*	−28.28 (13.39)	−19.38 (11.04)*	−26.08 (12.05)	−13.20 (9.84)*	−19.54 (12.90)	−8.23 (12.78)*	−16.66 (13.96)	−3.30 (11.14)*
3	P	12.67 (10.62)	11.46 (8.59)	24.74 (7.78)	17.00 (5.31)*	2.56 (11.18)	4,54 (8.04)	26.22 (12.92)	13.51 (11.90)*	19.80 (11.02)	11.25 (9.69)*
	S	−17.06 (7.16)	−12.15 (9.80)	−32.18 (4.67)	−33.77 (9.45)	−27.37 (8.59)	−23.69 (9.93)*	−21.93 (6.43)	−13.29 (9.63)*	−18.44 (8.39)	−9.05 (7.09)*
4	P	9.34 (30.79)	2.99 (23.87)*	13.95 (29.56)	8.88 (28.60)*	−0.63 (27.16)	−1.42 (26.43)	17.37 (33.27)	5.57 (26.40)*	12.71 (29.47 (	5.95 (24.59)*
	S	−27.29 (43.58)	−20.27 (41.29)*	−38.32 (28.29)	−34.66 (39.58)	−35.79 (36.39)	−26.38 (36.70)*	−35.18 (51.73)	−26.48 (51.92)*	−24.11 (39.41)	−20.26 (35.84)
5	P	16.98 (9.57)	12.75 (9.67)*	21.41 (12.67)	18.49 (11.26)	3.61 (12.79)	6.72 (10.20)	25.30 (11.68)	16.78 (10.97)*	19.01 (9.32)	15.48 (11.56)*
	S	−16.12 (9.30)	−12.45 (10.34)	−30.05 (6.28)	−24.90 (13.94)	−26.39 (8.77)	−20.88 (13.37)	−23.84 (8.78)	−15.84 (8.76)*	−16.93 (9.38)	−12.52 (11.80)
6	P	15.63 (12.47)	10.47 (6.62)	19.41 (11.11)	15.11 (8.77)*	6.17 (12.09)	1.20 (7.91)	23.71 (13.33)	12.07 (12.90)*	14.03 (12.21)	12.16 (7.90)
	S	−19.01 (8.83)	−15.96 (11.70)	−31.72 (6.86)	−36.08 (7.77)	−27.28 (5.34)	−30.81 (8.90)	−22.36 (8.00)	−22.84 (15.12)	−17.61 (10.63)	−19.58 (14.48)
7	P	14.99 (5.84)	6.89 (7.21)*	19.23 (8.78)	9.67 (8.06)*	3.06 (8.10)	−3.78 (6.91)*	23.91 (6.79)	8.49 (5.39)*	16.20 (4.65)	7.15 (8.61)*
	S	−21.02 (4.96)	−11.89 (5.89)*	−34.33 (9.14)	−21.57 (8.76)*	−28.34 (9.75)	−19.78 (6.37)*	−25.27 (4.80)	−13.04 (3.70)*	−19.81 (6.06)	−12.30 (5.74)*
8	P	14.86 (11.07)	14.39 (11.31)	19.09 (13.10)	20.17 (9.54)	1.66 (8.72)	2.40 (9.78)	22.74 (15.30)	18.08 (11.48)	15.83 (11.26)	14.99 (8.60)
	S	−22.79 (7.83)	−15.50 (9.10)*	−36.02 (7.28)	−35.55 (9.56)*	−31.86 (7.78)	−23.93 (13.12)*	−27.35 (9.31)	−22.04 (10.60)	−19.12 (8.26)	−13.69 (9.46)
9	P	23.15 (30.11)	22.29 (25.00)	29.81 (34.31)	25.75 (26.27)*	10.59 (29.83)	12.64 (22.90)	29.75 (31.77)	23.82 (30.08)*	26.09 (32.75)	23.23 (28.32)
	S	−14.33 (15.64)	−2.86 (21.68)*	−28.84 (22.23)	−22.03 (20.72)*	−22.18 (21.68)	−14.15 (21.06)*	−22.69 (20.82)	−9.29 (25.54)*	−10.41 (23.72)	−3.13 (21.96)*
10	P	20.38 (23.97)	17.68 (23.68)	27.84 (28.35)	22.92 (28.04)	7.16 (26.32)	9.12 (25.30)	30.89 (26.28)	22.24 (24.79)*	22.97 (24.83)	19.57 (25.36)*
	S	−11.64 (26.19)	−11.35 (25.75)	−23.03 (24.23)	−29.99 (21.45)	−19.63 (26.53)	−24.48 (21.09)	−18.35 (25.19)	−16.13 (29.73)	−14.73 (26.08)	−11.83 (25.04)

Statistical significance was obtained using Wilcoxon ranked tests (*α* = 0.05), with * and ** denoting *p* < 0.05 and *p* < 0.001, respectively, when with and without WHOs were statistically different. P, pronation; S, supination. The negative numbers imply supination.

#### Time to complete each task

Paired sample *t*-tests were used to assess the statistical differences between conditions for the time spent to complete each task, with statistical significance showing when the task took significantly longer with WHOs than without. The time difference and statistical significance are shown in Table 9 and Figure 6.

**Table 9:** Time difference (in seconds) for each ADL by WHOs calculated as with—and without WHOs.

WHO	ADL1	ADL2	ADL3	ADL4	ADL5	All ADLs
	Mean (SD) (s)	Mean (SD) (s)	Mean (SD) (s)	Mean (SD) (s)	Mean (SD) (s)	Mean (SD) (s)
1	−1.58 (1.21)*	−2.49 (1.50)*	−1.77 (2.22)*	−0.96 (3.27)	−0.39 (0.99)	−1.36 (1.99)*
2	−1.30 (1.04)*	−2.15 (0.98)**	−0.52 (1.64)	−0.70 (2.16)	−0.86 (1.60)	−1.22 (1.39)*
3	−0.74 (2.18)	−1.62 (2.02)*	−1.72 (1.23)**	−0.01 (3.34)	−0.40 (2.59)	−1.09 (1.89)
4	−0.67 (1.13)*	−5.91 (2.56)**	−3.88 (1.64)**	−0.03 (2.19)	−1.04 (1.59)*	−0.88 (2.62)*
5	−1.16 (0.51)**	−2.13 (1.85)*	−2.49 (0.90)**	−0.27 (1.57)	−0.82 (0.98)*	−1.08 (1.35)**
6	−0.71 (1.34)	−0.79 (1.22)*	−1.01 (1.65)	−0.28 (2.70)	−0.20 (0.72)	−0.95 (1.11)**
7	−1.36 (1.53)*	−3.37 (2.52)**	−1.16 (1.65)*	−0.03 (1.72)	−0.61 (0.80)	−0.98 (1.96)
8	−0.57 (1.22)	−1.38 (1.54)*	0.01 (3.89)	−0.13 (2.77)	−0.22 (0.70)	−1.00 (1.48)*
9	−0.76 (1.36)	−1.55 (1.46)*	−0.81 (1.60)	0.02 (3.54)	−0.19 (1.49)	−1.81 (1.85)
10	−0.55 (0.85)*	−0.95 (1.29)*	0.10 (1.32)	0.72 (1.76)	−0.80 (0.76)	−2.14 (1.18)**

Statistical significance was obtained using paired-sample *t*-tests (*α* = 0.05), with * and ** denoting *p* < 0.05 and *p* < 0.001, respectively, when the task took a significantly longer time with WHOs than without WHOs. The negative numbers indicate that the condition with WHO is longer.

As highlighted in Table 9, all WHOs negatively impacted the mean time across all participants when completing ADL1 and ADL2, with most having a significant (*p* < 0.05) or highly significant (*p* < 0.001) detrimental impact. ADL2, in particular, was adversely affected by WHO use. Conversely, the other two ADLs, particularly ADL4, were much less affected by WHO use, although a trend could be observed in which the use of WHOs increased the task completion time. When the overall time was assessed across ADLs, the best performing WHO was #10. The worst performing WHO was #4, with ADL1, 2 3, and 5 taking a statistically longer time than without the WHO condition (Table 9).

#### Volar bar angle

The volar bars did not deform or change shape throughout the testing period for any of the participants.

## Discussion

Prefabricated WHOs are prescribed for a range of conditions, with some prescribed for night use only, whilst others should be worn during the day to aid function and promote independence. These functional objectives should be achieved through a realignment of the wrist/hand and motion control of the wrist whilst facilitating grip patterns. However, over the last few decades, prefabricated WHOs have remained essentially unchanged, and this research challenges the efficacy of current designs.

Previous research demonstrated, during range of motion testing, that whilst these 10 WHOs may provide some reduction in wrist flexion, extension, radial, and ulnar deviation, none successfully nor consistently immobilised the wrist, and crucially, none prevented movement into flexion (20). Further to this, these WHOs negatively impacted grip strength, with the wrist often adopting an abnormal position to achieve maximal grip, and it was hypothesised that this could have happened due to their poor design, especially in the palmar area, which negatively impacted grip strength (20). This detrimental impact can directly make some tasks difficult, dangerous, and time-consuming to do, with the wrist adopting abnormal positions, which may exacerbate pain. If when wearing a WHO, an adequate grip strength can be achieved only with the adoption of abnormal wrist positions, it raises important concerns, particularly for those users who have underlying conditions that are degenerative and progressive in nature. To inform prescription practices, clinicians need to understand whether WHOs maintain the wrist in the desired position during ADLs, understand their impact on time to perform ADLs, and whether there are specific design features that could adversely impact the ability to perform ADLs.

Of note, there was no significant change in any participant's weight during the 10 weeks of testing that could have influenced the results. Similarly, there was no change to the angle of the volar bar on the completion of each test, and consequently, this did not impact the results.

### Interpretation

#### Motion control

The resulting mean resting position in the sagittal plane with WHOs across all participants, as shown in Table 2 and Figure 2, ranged from 1.25° extension (± 6.05°) for orthosis #4 to 8.87° extension (± 5.13°) for orthosis #6, with WHO#1, 2, 3, 6, and 9 positioning the wrist within the desired prescribed range of [10–15]° extension. Similar to previous research, each WHO was fitted as supplied by the manufacturer (20), with no contouring of the volar bar to alter the alignment of the wrist where this was an option to do so (WHO#1, 2, 3, 5, 6, 7, 8, and 9). These results emphasise that clinicians must not assume that manufacturers provide these WHOs with a volar bar contoured to hold the wrist at a suitable angle at rest and should be adjusted if possible.

In the coronal plane, the resulting mean resting position with WHOs across all activities ranged from 3.43° (±3.62°) of ulnar deviation for WHO#9 to 10.41° (±6.77°) of radial deviation for WHO#3, with only WHO#9 preventing an undesirable resting position of radial deviation (Table 2 and Figure 2).

Variability across the mean resting position was observed in the transverse plane with WHOs across all activities, with an observable trend towards a position of supination (Table 2 and Figure 2).

### Wrist movement during each task

#### Flexion

Flexion control is one of the most important motions to be controlled when functional WHOs are prescribed for people presenting with wrist/hand dysfunction or utilised as personal protective equipment in the workplace or during recreational activities.

As seen across all ADLs without WHOs, the mean maximum flexion angle is task-dependent, with ADL4 (zipping a jacket) being the activity that has been shown to require the most degrees of flexion to complete the task. All other tasks, in particular, ADL1, 3, and 5 require a stable wrist position to be attained in the sagittal and coronal planes throughout the activities.

Across all WHOs and ADLs, an overall reduction in the mean maximum flexion angle and the percentage of time spent in flexion was observed when compared with that without the WHO condition. However, in nine out of ten WHOs, and for at least one ADL for each WHO, the wrist was flexed for a significant amount of time, with only WHO#1 successfully reducing flexion whilst allowing no significant amount of time in this arc of motion across all activities.

For ADL1 (pouring water from a jug), maximum wrist flexion with all WHOs was significantly less in comparison with that without WHOs, with only WHO#1 (*p* < 0.001) and WHO#9 (*p* < 0.05) adopting a neutral or extended wrist position. When the time spent in flexion for this task was analysed, WHO#1 showed the least time in flexion [1.17% (±3.52)], with little variability across participants, unlike WHO#9, which, although significantly reduced the time in flexion, had an increased variability through this activity and across participants.

For ADL2 (turning a key in a lock), a task that predominantly requires pronation/supination, all WHOs (but #3) managed to successfully reduce the mean maximum flexion angle, with only WHO#2 and #5 significantly restricting it (*p* < 0.05). This is observed to be in line with the percentage of time spent in flexion (Table 4).

Maximum flexion with and without WHOs during ADL3 (cutting putty with a knife) was significantly reduced for all WHOs, except for WHO#1; however, no WHO was found to restrict it during this task. The mean percentage time in flexion with WHOs was observed to range from 0.86% (±2.16) for WHO#2 to 13.82% (±23.73) for WHO#4.

For ADL4 (zipping a jacket), without a WHO, the wrist was observed to spend a considerable percentage of time in a position of flexion, which may present a challenge to undertaking the activity with a WHO designed to prevent wrist flexion. If wrist flexion is successfully blocked, compensatory motion such as internal rotation of the shoulder would be required. Across all WHOs, there was a significant reduction in wrist flexion, but importantly, there was no WHO that successfully blocked this movement throughout the activity. When the mean percentage time in flexion with a WHO was considered, the range was from 6.88% (±11.98) for WHO#1 to 32.48% (±28.27) for #10. Interestingly, and unsurprisingly, WHO#1 and #3 performed best at reducing the time spent in flexion; these two WHOs are identical, except for an additional circumferential wrist strap on #3. These WHOs are 23 cm in length, constructed from a two-way stretch fabric with Velcro® fastenings and an aluminium volar bar. In line with previous research investigating the efficacy of these 10 WHOs in controlling wrist motion, it was concluded that the same WHOs (#1 and 3) performed better in controlling wrist flexion, which was attributed to the improved biomechanical efficiency related to the increased length of these WHOs and the ability to achieve a good fit due to the material of construction (20). In addition, this research has demonstrated that in addition to the aforementioned, the addition of a wrist strap provides enhanced flexion control through activities that require the wrist to be in flexion.

For ADL5 (carrying a laden plate), the mean maximum flexion for all WHOs was reduced when compared with that without a WHO condition. Five out of ten WHOs (#1, 2, 3, 5, and 9) successfully blocked flexion during the activity, with no test scenario having wrist flexion over a significant amount of time with the WHOs. However, nine out of ten had a significant reduction in the percentage of time in which the wrist was in flexion when compared with a no WHO condition.

Of importance, when all ADLs were considered for all WHOs across all participants, no WHO was able to successfully and consistently block wrist flexion (Figure 3). When the mean percentage of time spent with the wrist flexed was assessed across all ADLs for each WHO, it could be observed that there was important variation in the data across participants (as highlighted by the outliers in Figure 3), with some WHOs allowing some activities to be carried out in a flexed position >80% of the time (WHO#4, 7, 8, 9, and 10). Interestingly, WHO#4 represents a typical WHO that circumferentially encompasses the hand, wrist, and forearm with high durometer silicone, and as such, good motion control would be expected. However, as highlighted in the previous work, the poor ability to achieve an optimal fit due to the lack of adjustability with this WHO may mean that there is a poor ability to control wrist flexion (20). Although WHO#6, 8, and 10 were observed to enable a more optimised circumferential fit due to the fabric of construction, these were shown to have poor wrist flexion control. Two of these, at 18 cm in length (WHO#6 and 10), were the two shortest WHOs tested. This is in line with previous findings where from a biomechanical efficiency perspective, a longer WHO may better control wrist flexion motion (20).

#### Extension

Positioning the wrist between 10° and 15° of extension, whilst enabling finger movement, has been shown to facilitate hand function during day-to-day activities (11, 12). However, when the wrist is positioned at greater than 15° of extension, it may have a negative impact on carrying out many ADLs. During our study, and similar to other movements, extension control was found to be highly task-dependent, with all WHOs allowing over 15° extension for significant periods of time. Unsurprisingly, ADL4 (zipping a jacket), which requires a significant amount of time to be spent in wrist flexion, was shown to have less percentage of time spent in extension in comparison with other ADLs. This is attributable to the task rather than the ability of the WHOs to limit the time spent in extension. ADLs 1, 3, and 5 require a stable wrist (10°–15° extension) and grip to be maintained throughout activity with WHOs. A statistically significant percentage of time above the desired position of 15° extension was observed for all WHOs in ADL1 and for all WHOs except for #4 during ADL3 and ADL5. The current designs of WHOs typically do not integrate specific design features, such as dorsal stiffness, which could restrict wrist extension. WHO#4, which fully encompassed the hand, wrist, and forearm with high durometer silicone, was the most rigid in the dorsal aspect, and as such, appeared to have had better extension control in comparison with the other devices. This finding correlates with previous research (20). When extension control was assessed across all ADLs and participants, all WHOs were observed to allow a range of extension greater than 15° for a significant amount of time.

#### Radial deviation

Specifically for those with RA, blocking wrist radial deviation is desirable. Moreover, there is strong research evidence that the maximum power grip strength is achieved with the wrist in a position of extension between 15° and 30° (8, 9) with a position of up to 15° of ulnar deviation in the coronal plane (8, 10). However, only WHO#9 during ADL4 provided some level of restriction of radial deviation, but this was not consistent across all participants. All participants were found to spend a significant amount of time during the tasks with their wrists in a radial deviation. As such, none of the WHOs tested had design characteristics that could address the functional requirement of preventing radial deviation.

#### Combination of flexion with ulnar deviation

In addition to being detrimental to power grip, the combination of flexion and ulnar deviation is also associated with increasing levels of pain (6). As observed in Table 7, and as for all the other motions, the efficacy of the WHOs is highly task-dependent. In particular, it was expected that ADL4 (zipping and unzipping a jacket) would require participants to adopt this position the most, and when this activity was observed with the WHOs, the percentage of time spent in flexion and ulnar deviation was least with WHO#1, 3, and 8. This correlates with the flexion restriction afforded by WHO#1 and 3 during ADL4 when analysing flexion control independently. WHO#7 and 9 were shown to unsuccessfully block the combination of these two wrist movements. When all the ADLs were compared by WHOs across participants, 4 out of 10 WHOs (#5, 7, 9, and 10) were found to block these movements less than when assessed for individual tasks, which further highlights the great variability and lack of efficiency and effectiveness of the WHOs to work consistently regardless of the activity undertaken.

#### Pronation/supination

Because of the limited evidence to inform the specific limitations of pronation and supination, the focus of this work was to quantify it and to report the impact that WHOs had on movement in the transverse plane during ADLs, as reported in Table 8. WHO#2 and 7 were seen to have the most statistically significant reduction for both pronation and supination, followed by WHO#4, and WHO#6 and 10 had the least impact on the reduction of the range of motion. At 18 cm, these two WHOs (#6 and 10) were the shortest of all devices tested, so it may be possible that the reduced length, in combination with the flexibility of the construction materials (two-way stretch fabric and neoprene, respectively) and impact of the low-impact strapping, may be responsible. Conversely, the three WHOs with improved motion control are amongst the longest, being 23 cm (#2) or 25 cm (#4, 7) in length. Other WHOs such as #5 performing in the mid-range of providing motion control, in addition to having a volar bar, have unique plastic dorsal stays associated with the strapping, which contribute to the overall stiffness of the WHO and could positively impact further motion restriction. Similarly, WHO#4, which is one of the longest at 25 cm in length, circumferentially contains the hand, wrist, and forearm in high durometer silicone, which would contribute to transverse plane control.

As mentioned previously, there is currently limited evidence that may allow one to make inferences in the data regarding the restriction of movement in the transverse plane. Figure 6 represents the data from a single participant chosen at random, highlighting a typical example of the execution of the tasks assessing pronation and supination with and without WHO#2. This WHO was chosen as an example, as this was the one that was shown to have the greatest impact on the transverse plane motion. It can be further observed that, during ADL1 (pouring water from a jug), the peaks of pronation and supination correspond to the tilting of the jug in readiness to begin and complete the pour, which can be observed to be controlled well by WHO#2. Turning a key in a lock (ADL2) is a task involving repetitive arcs of pronation and supination, and as further observed in Figure 6, as the amplitude of the movement is reduced as a result of the WHO, the number of peaks and valleys (representing pronation and supination) and the width of these and overall time to complete the task also increase. Although pronation/supination is not a movement normally associated with cutting/chopping, it was observed to be present during ADL3 and was associated with the period of time in which the participant repositioned the knife on the putty in preparation to make the second cut. Whilst WHO#2 could be observed to control this unexpected motion reasonably well, the participant took a significantly longer time to cut through the putty with the WHO restricting this motion than without. This could also be attributed to the fact that the participant was less able to achieve the required grip pattern due to the presence of the ill-contoured volar bar. When ADL4 was analysed, zipping and unzipping a jacket required some pronation/supination, as the zip was raised and then lowered. This task was adequately controlled by WHO#2 (Figure 6) with minimal impact on the time taken to complete the task. Finally, during the period of time when the laden plate was moved (after the laden plate was grasped and subsequently released during ADL5), minimal pronation/supination was required, which could be observed to be well controlled by WHO#2.

#### Time to complete a task

The effect that each WHO had on the time to complete a task can have a large cumulative effect across all tasks throughout a day. Minimising the detrimental effect of a WHO may increase adherence. It can be considered that a WHO that increases task time will directly increase the time that the wrist/hand may be under load during a task, which may further exacerbate existing pain or injury.

Some factors that could influence the time to complete a task are interrelated design features. The WHOs that better restricted the wrist motion required to complete a task negatively impacted the time taken. Similarly, WHOs that impact the ability to adopt the required grip pattern can adversely influence the time to complete a task. For example, a power grip where the fingers flex towards the palm can be negatively affected by a WHO that encompasses the palm of the hand and is poorly contoured in this area (20). Also, if the palm section of the WHO is made of an unyielding material that will not adequately conform to the shape of the object to be gripped, poor contact between these two surfaces will result in an unstable grip that will impact a person's ability to perform a task safely.

The tasks involving a power volar grip were ADL1 and ADL3 (pouring water from a jug and cutting putty, respectively). These activities required the WHO to maintain the wrist in the desired position without adversely affecting the required grip pattern. All the WHOs increased the time to complete ADL1, with 6 out of 10 (#1, 2, 4, 5, 7, and 10) reaching statistical significance. All the WHOs tested had a design of a volar bar that encroached into the palmar hand region, which could have impacted the grip patterns adopted. All the WHOs, except #4 and #9, had identical designs of volar bars with the same lack of contouring into the transverse palmar arch, which could have impacted the ability to perform this ADL. In contrast, WHO#6, 8, and 9 at 18 and 20 cm in length were some of the shortest WHOs, and as shown in the range of motion results, the length of the device influenced motion control. With these shorter WHOs that have poorer motion control, it is possible that to achieve a secure grip on the jug with a poorly contoured palm section, wrist motion, fixed wrist/hand positions, and alternate grip patterns (not usually required for the task) can be easily adopted to facilitate a secure grip. This, in turn, would allow the task to be completed with less impact on time than with longer WHOs with associated improved motion control but identical volar bars and poor contouring into the transverse palmar arch. WHO#9 has a volar bar and palmar section with improved contouring and may account for a reduced impact on time. WHO#4, which has a different but equally poorly designed volar bar, also has a very poorly contoured palmar area and was found to increase the time to complete the task significantly. WHO#3 also increased the time to complete ADL1 but was not statistically significant. This 23 cm WHO has been shown to provide optimal wrist flexion control in comparison with the other WHOs. It also has the same poorly contoured volar bar that most of the WHOs have but with the notable difference of being the only WHO that has an additional circumferential wrist strap that maintains the volar bar in a good position during activity. It could be that the increased length and subsequent motion control, in combination with the volar bar held firmly in position, provided the most optimal design for this task. Most WHOs increased the time to complete ADL3, which required a diagonal volar grip, and was most adversely impacted by five of the WHOs, with #4, 5, and 3 having a greater time increment when compared with without WHO, respectively. As detailed previously, WHO#4 was shown to have the worst palmar contouring, which would directly impede the required grip pattern and adversely impact the time required to complete the task.

ADL2 (turning a key), which involves a key grip, predominantly requires a movement of pronation/supination, and therefore, the time to complete the task would be most adversely impacted by a WHO optimally controlling pronation/supination. WHO#2, 4, and 7, which were shown to best control pronation/supination, also had the most negative impact on the time required to complete this task. WHO#4 (25 cm), which completely circumferentially contains the forearm, wrist, and hand, is made from a high durometer silicone device that stops just proximal to the metacarpal-phalangeal joints and was found to impede movement in the transverse plane. The other two WHOs [#2 (23 cm) and #7 (25 cm)], which also circumferentially contain the forearm, wrist, and hand, are constructed from either neoprene (synthetic rubber) or a knitted two-way stretch elasticated material, respectively. Although these fabrics of construction enabled a greater fit to be achieved, these flexible materials provided less resistance to pronation/supination, and therefore, had slightly less impact on the time taken to complete the task when compared with WHO#4. When all ADLs were assessed together, all WHOs had a negative statistical impact on the time to complete the tasks.

ADL4 (using a zipper) typically requires the wrist to adopt a position of flexion and needs minimal additional wrist motion during activity, being mainly reliant on elbow motion. As such, if the wrist can be held in a pain-free position, the time to complete the ADL should not be adversely affected, as shown in the results. However, a WHO that prevents movement into wrist flexion may require an additional internal rotation of the shoulder, which may be problematic in the presence of systemic disease.

Similarly, ADL5 (a laden plate) requires minimal wrist motion during the task but does require the wrist to be stabilised in a pain-free position (control of sagittal and coronal plane motion) with the maintenance of a lateral grip, and as such, should have little impact on task time, as is seen in the results.

When the results across all 10 WHOs and ADLs are considered, aspects that may influence these can be compared. There is some evidence in the results that suggests that there are many interrelated factors, including the length of the WHO, quality of fit, materials of construction, and palmar contouring that influence the results. Variations in the performance between the WHOs tested might be a contributing factor for the current conflicting evidence pertaining to WHO functionality across tasks and participants and indicates that there is a capacity to modify their design to better address the orthotic management objectives.

In previous research, shorter WHOs (18 cm and 20 cm) #10, 8, 6, and 9 were shown to provide reduced wrist control (20), and as such, the time required to complete tasks might not be adversely impacted as a result of this, as demonstrated in the results. In contrast, longer orthoses have been shown to provide enhanced wrist motion control, with materials of construction also fundamental to functionality (20), and in this research, longer WHOs (25 cm) #4, 2, 3, and #7, which have been shown previously to better resist wrist motion, showed an increased detrimental impact on time to complete the ADLs. The outlier to these results is #5, which is the shorter version of #7 (20 cm in comparison with 25 cm) but ranks higher in adversely impacting the time to complete the ADLs.

Across all ADLs, WHO#4 had the greatest negative impact on the time to complete tasks and also had the poorest contouring in the palmar section. All other WHOs other than #9 and #3 had similar designs of volar bars, and so, it is hypothesised that a difference in the time to complete the tasks between these WHOs is not attributed to this design feature. It was observed that whilst WHO#3 had the same volar bar as most of the others, this volar bar was better secured in position by an additional circumferential wrist strap that appeared to have reduced the negative impact on the time taken to complete the tasks.

Integrated stiff structures held firmly in place and that are located in the anatomical planes in which motion should be restricted, must be present. This research has also shown that the volar bar that is designed and positioned to restrict wrist flexion is poorly designed at the distal aspect that extends into the palm of the hand. This poor contouring not only can negatively impact the ability to achieve some grip patterns, but the presence of unyielding contact surfaces in this area of the orthosis can make gripping of rigid objects precarious and may require the adoption of abnormal and compensatory joint positions of the wrist and the more proximal joints of the upper limb. The strap design and strap positioning are hypothesised to contribute to enhanced motion control, and maintaining the volar bar in position should not be considered simply a way of retaining the WHO on the arm. Although in this study, all WHOs were fitted by the same clinician to each participant to optimise fit and function, most wearers don these devices independently. Hence, the strap design and position will significantly impact the user's ability to put a WHO on and fasten it, often using only one hand to do so. As the user’s difficulty in achieving this may impact the quality of fit, comfort, and functionality of the WHO, further research to investigate this aspect is required.

### Study limitations

Only healthy participants were recruited for this study, as patients with RA and many other conditions are likely to experience pain that may limit wrist motion and the ability to perform ADLs. This was done so that the biomechanical efficacy of the WHOs could be tested without the influence of confounding factors.

A greater sample size would allow for making an additional comparison of the WHOs. However, given the high number of repetitions presented in this study, and its comparative nature, the authors would not expect a greater sample size to lead to different results. In addition, with the evidence presented, future research should calculate an appropriate sample size and power based on the anticipated effect sizes.

The Biometrics DataLOG unit and goniometers were subject to accuracy uncertainties, with ±2° listed in the specifications for the goniometers (28). However, to minimise positional uncertainties when placing the electro-goniometers, each unit was checked using a traditional mechanical goniometer.

An additional limitation could relate to the fit of the prefabricated WHOs. Although the participants were fitted with a WHO appropriately sized for their wrists in line with the manufacturer's sizing guidelines, it does not guarantee an optimal fit. To overcome this drawback, once fitted and with appropriate tensioning of the straps, a further visual assessment of fit was made as is done in normal clinical practice by the expert clinician. If this was deemed to be acceptable, testing was undertaken. Importantly, there are no known methods to accurately quantify the quality of fit of a WHO; hence, errors in the results pertaining to fit could not be quantified.

As all WHOs were fitted by the research team, as opposed to the participants, the best fit for each WHO was achieved, thus demonstrating the maximum potential efficacy of each WHO as a result of the design characteristics. However, in practice, a WHO is fitted by individuals themselves who would be unlikely to consistently and repeatably achieve this optimal fit using their contralateral hand, which may also present with dysfunction. As such, this research demonstrates the optimal efficacy of WHOs, which may not be achievable in a patient group when independently donned.

The shoulder joint was not instrumented to measure the range of motion, and as such, there was no ability to quantify any compensatory movements that could have been required to facilitate the undertaking of each ADL with WHOs. In particular, it is thought that the restriction of pronation/supination during tasks typically requiring these motions (such as ADL2) would necessitate increased shoulder rotation. Similarly, although the elbow was instrumented to identify the start/end of each ADL, there was no analysis of elbow flexion/extension range of motion, which could also have been altered to facilitate the completion of ADLs with WHO use.

As the results show that WHO impact is task-specific, additional ADLs may have demonstrated further effect.

## Conclusions

In summary, clinicians, when prescribing a prefabricated WHO, should consider the required orthotic objectives and select an orthosis based on length, materials of construction, strapping configuration, contouring of the palmar section, and the ability to customise the fit. Whilst these parameters affect functionality, clinicians providing optimised patient-centric rehabilitation must acknowledge that, whilst research has shown that WHO wearers wish to have devices that provide more support, reduce pain, and improve the ability to carry out ADLs, factors such as bulkiness and poorly fitting, uncomfortable WHOs that are easily soiled negatively impact adherence (29). Finally, it has been shown that there are significant patient concerns regarding poor aesthetics, difficulty in independently putting on/taking off the WHO, and the reliance on Velcro™ fastenings that damage clothing. As such, clinicians must consider all these aspects of design when prescribing a WHO. Future designs of WHOs should address these concerns and improve efficacy and functionality, thereby positively impacting the quality of life for the wearer of these devices, regardless of the underlying condition and health economics.

To further inform evidence-based practice, in addition to developing robust methodologies, it is highly recommended for researchers to consistently report precise information about their study, including a detailed description of the methodology, participant characteristics, and the design of orthoses tested. Repeatable and clinically relevant research positively impacts patient care. The authors recognise the requirement for further research into the use of “functional” prefabricated WHOs to evaluate the long-term impact of use on grip strength, grip endurance, grip patterns, additional activities of daily living, and pain in different populations to further understand their functionality. In addition, the efficacy of WHOs should be further tested with participants presenting with wrist/hand dysfunction donning their own orthoses rather than WHOs being optimally fitted by a clinician.

## Data Availability

The raw data supporting the conclusions of this article will be made available by the authors, without undue reservation.
